# Supplementary material for: A Critical View of the Application of the APEX Software (Aqueous Photochemistry of Environmentally-Occurring Xenobiotics) to Predict Photoreaction Kinetics in Surface Freshwaters
Source: Molecules. 2019 Dec 18;25(1):9. doi: 10.3390/molecules25010009 (PMC7017383; doi:10.3390/molecules25010009)
Supplement: Supplementary file 1 [file molecules-25-00009-s001.zip › 3.2.4_gcc-4.4.0.zip!/3.2.4_gcc-4.4.0/3.2.4_gcc-4.4.0/doc/octave/html/interpreter/Bug-Criteria.html]

Bug Criteria - Untitled


Next: Bug Lists,
Previous: Reporting Bugs,
Up: Trouble


---

### E.3 Have You Found a Bug?

If you are not sure whether you have found a bug, here are some guidelines:


- If Octave gets a fatal signal, for any input whatever, that is
  a bug. Reliable interpreters never crash.

  - If Octave produces incorrect results, for any input whatever,
    that is a bug.

    - Some output may appear to be incorrect when it is in fact due to a
      program whose behavior is undefined, which happened by chance to give
      the desired results on another system. For example, the range operator
      may produce different results because of differences in the way floating
      point arithmetic is handled on various systems.

      - If Octave produces an error message for valid input, that is a bug.

        - If Octave does not produce an error message for invalid input, that is
          a bug. However, you should note that your idea of “invalid input”
          might be my idea of “an extension” or “support for traditional
          practice”.

          - If you are an experienced user of programs like Octave, your suggestions
            for improvement are welcome in any case.
